# Histomorphological Changes in the Canine Epididymis During Downregulation and Recovery After Deslorelin Treatment

**DOI:** 10.1111/rda.70013

**Published:** 2025-02-12

**Authors:** Henriette Greiner, Hanna Körber, Eva‐Maria Packeiser, Ines Focks, Selim Aslan, Serhan Ay, Murat Findik, Gülşah Saral, Eser Akal, Christelle Fontaine, Sandra Goericke‐Pesch

**Affiliations:** ^1^ Unit for Reproductive Medicine—Clinic for Small Animals University of Veterinary Medicine Hannover, Foundation Hannover Germany; ^2^ Department of Obstetrics and Gynaecology, Faculty of Veterinary Medicine Near East University Nicosia Cyprus; ^3^ Department of Obstetrics and Gynaecology, Faculty of Veterinary Medicine Ondokuz Mayıs University Samsun Turkey; ^4^ Department of Reproduction and Artificial Insemination, Faculty of Veterinary Medicine Ondokuz Mayıs University Samsun Turkey; ^5^ Global Marketing and Market Development Virbac Group Carros France

**Keywords:** castration, dog, epididymis, slow‐release GnRH agonist

## Abstract

Slow‐release GnRH agonist implants containing deslorelin (SRI) are registered for temporary suppression of male fertility. The effect of SRI treatment on canine testicular function is well characterised, although the effect of downregulation and subsequent recovery on epididymal function has not been studied yet. Therefore, twenty‐nine healthy male dogs were treated with a 4.7 mg SRI for five months. Subsequent to implant removal, groups of 4–5 dogs were surgically castrated either at implant removal (week 0) or 2, 4, 6, or 10 weeks later. Three subgroups were categorised according to pre‐surgical testosterone levels. Five healthy untreated dogs served as control. Epididymides were separated into head, body and tail. Epididymal duct diameter and epithelial height were measured using haematoxylin–eosin‐stained sections of each dog and part of epididymides. Besides, the presence of spermatozoa, the cilial height, the thickness of the muscle layers and the relative amount of connective tissue were semiquantitatively assessed. The downregulated epididymis was characterised by a reduced epithelial height and epididymal duct diameter, lower cilia and absence of sperm, but more connective tissue, supporting that epididymal function is significantly altered by SRI treatment. At recovery subsequent to implant removal, the histomorphology was comparable with untreated controls. The study indicates that recovery of the epididymal function, like spermatogenesis, depends on testicular testosterone production.

## 1 Introduction

The interest in alternatives for surgical castration in male dogs has increased significantly in the last two decades (Stempel et al. 2022a). Besides the well‐known short‐term risks of anaesthesia and immediate surgical and post‐surgical complications, the awareness of possible long‐term side effects has significantly increased among veterinarians and dog owners (Downes et al. 2015). Meanwhile, numerous studies on possible consequences of androgen withdrawal and altered gonadotropin secretion after surgical castration emphasise the rising research interest. Post‐castration side effects include obesity (Bjørnvad et al. 2019), orthopaedic problems (Kutzler 2020) and tumour predispositions (Goericke‐Pesch 2017; Hart and Hart 2021; Root Kustritz 2012). Besides, the irreversibility of surgical neutering is without a doubt not only threatening to breeders but also to pet owners who consider breeding‐use in the future and those questioning the benefits of surgery, for example, in case of behavioural problems. Temporary suppression of gonadal function by slow‐release GnRH‐agonist implants (SRI) has been described to be successful for non‐surgical fertility control, but also as a test castration. Various GnRH‐agonist SRIs, containing buserelin (Goericke‐Pesch et al. 2010; Riesenbeck et al. 2002), azagly nafarelin (Goericke‐Pesch et al. 2012, 2009; Ludwig et al. 2009) or deslorelin (Junaidi, Williamson, Martin, et al. 2009; Junaidi, Williamson, Trigg, et al. 2009; Junaidi et al. 2003, 2007; Trigg et al. 2001), have been researched in detail, and the effect of treatment‐related testosterone withdrawal on canine testicular function is well characterised.

However, the effects on epididymal function have so far been neglected, although the epididymis is crucial for male fertility and has an important meaning for reproduction. The canine epididymis is, like the epididymis of other species, attached to the testis and macroscopically divided into three segments: the head, the body and the tail. The epididymal transit takes around 10 to 14 days (Ali Hassan et al. 2021). The head and body are responsible for the secretion of the epididymal fluid containing specific proteins, such as CRISP I (Cohen et al. 2000), P26h (Légaré et al. 1999) or Eppin (Richardson et al. 2001), to ensure successful sperm motility, maturation and fertilising capacity (Ali Hassan et al. 2021; Belleannée et al. 2012; Cornwall 2009; Turner 1995). The epididymal tail serves as a storage reservoir for mature, fertile spermatozoa (Ali Hassan et al. 2021; Nicander 1971).

We postulate that the hormonal changes during so‐called downregulation affect epididymal histology and subsequent function. To better understand the effects of deslorelin‐containing SRIs on canine epididymal function, we aimed to investigate morphological characteristics of the different regions of the epididymis at downregulation and during subsequent recovery and compare them to healthy untreated controls.

## 2 Material and Methods

The Animal Experiments Local Ethics Committee of Ondokuz Mayis University approved animal experimentation (approval number: 2015/52). A detailed description of the animals and the experimental design were previously published (Vasetska et al. 2023).

### 2.1 Animals

Twenty‐nine healthy mature pure (*n* = 15) or mixed‐bred (*n* = 14) male dogs of 8 different breeds with a mean age of 20.8 ± 7.5 months (range: 12–36 months) and a mean body weight of 20.0 ± 5.3 kg (range: 10–35 kg) were included in the study. They were randomly assigned to a treated group (TG) and an untreated control group (CG). The dogs belonging to the CG (*n* = 5) were all cross‐breed, with a mean age of 17.6 ± 4.8 months (range: 12–24 months) and a mean body weight of 20.2 ± 3.5 kg (range: 16–25 kg). The dogs belonging to TG (*n* = 24) were aged 21.5 ± 7.9 months (range: 12–36 months) with a mean body weight of 20 ± 5.7 kg (range: 10–35 kg).

### 2.2 Experimental Design

In the CG dogs, a general and andrological examination was performed, and a blood sample for testosterone measurement was taken prior to the surgical castration.

Following an initial examination and blood sampling for testosterone analysis, the TG dogs were treated subcutaneously with a 4.7 mg slow‐release implant (Suprelorin, 4,7 mg; Virbac, France) into the paraumbilical area. Five months after treatment, the SRI was removed under local anaesthesia. Andrological examinations and blood samplings were performed before the removal. Groups of four to five dogs were surgically castrated at different time points: week 0 (5 months after treatment = implant removal), 2, 4, 6 and 10 after implant removal. The epididymes were collected and preserved for further investigations. Another blood sample for testosterone measurement was taken right before the castration (Vasetska et al. 2023). Testosterone values of ≤ 0.1 ng/mL were considered basal. Based on the testosterone concentrations at castration, TG was divided into three groups: TG1 with testosterone ≤ 0.1 ng/mL, TG2 with testosterone ranging between 0.2 and 0.5 ng/mL and TG3 with testosterone > 0.5 ng/mL.

### 2.3 Processing of the Epididymal Tissues and Histomorphological Assessment

Following separation into the three segments (head/body/tail) and trimming of tissue cubes of about 1 cm^3^, the tissue was fixed in Bouin's solution at 4°C for 24 h (Goericke‐Pesch et al. 2009), washed repeatedly with 70% ethanol and paraffine‐embedded. Sections (2–3 μm) were cut, haematoxylin–eosin stained and mounted with Roti Histokitt (Carl Roth GmbH + Co. KG, Karlsruhe, Germany) as previously described (Goericke‐Pesch et al. 2009).

The histomorphological assessment was performed with the evaluator being blinded to the dog, treatment group, and epididymal segment to avoid any related bias during evaluation. One section for each dog and segment was evaluated in total with a light microscope (Zeiss West, Oberkochen, Germany). Besides a general overview and histological evaluation, the following parameters were investigated: epithelial height and epididymal duct diameter (both quantitatively), presence of spermatozoa within the duct, cilial height, relative amount of connective tissue, and thickness of the muscle layers (all semiquantitatively).

For measurements of the epithelial height and total diameter of the epididymal duct, photographs of the slides were taken at a 40‐fold magnification by using an Olympus BX41TF Microscope (Olympus, Tokyo, Japan) with an Olympus DP72 camera (Olympus Corporation, Tokyo, Japan) and the Olympus cell Sens Dimension Software (version 2.1, Olympus Corporation, Tokyo, Japan). Image J (1.54d, Wayne Rasband and contributors, National Institutes of Health, MD, USA) was used for measurements. In every dog and segment, twenty nearly round epididymal duct sections were measured for their total and lumen diameter at two different localisations. In order to determine the epithelial height, the diameter of the lumen was subtracted from the total diameter, and the difference was divided by two.

**FIGURE 1:**
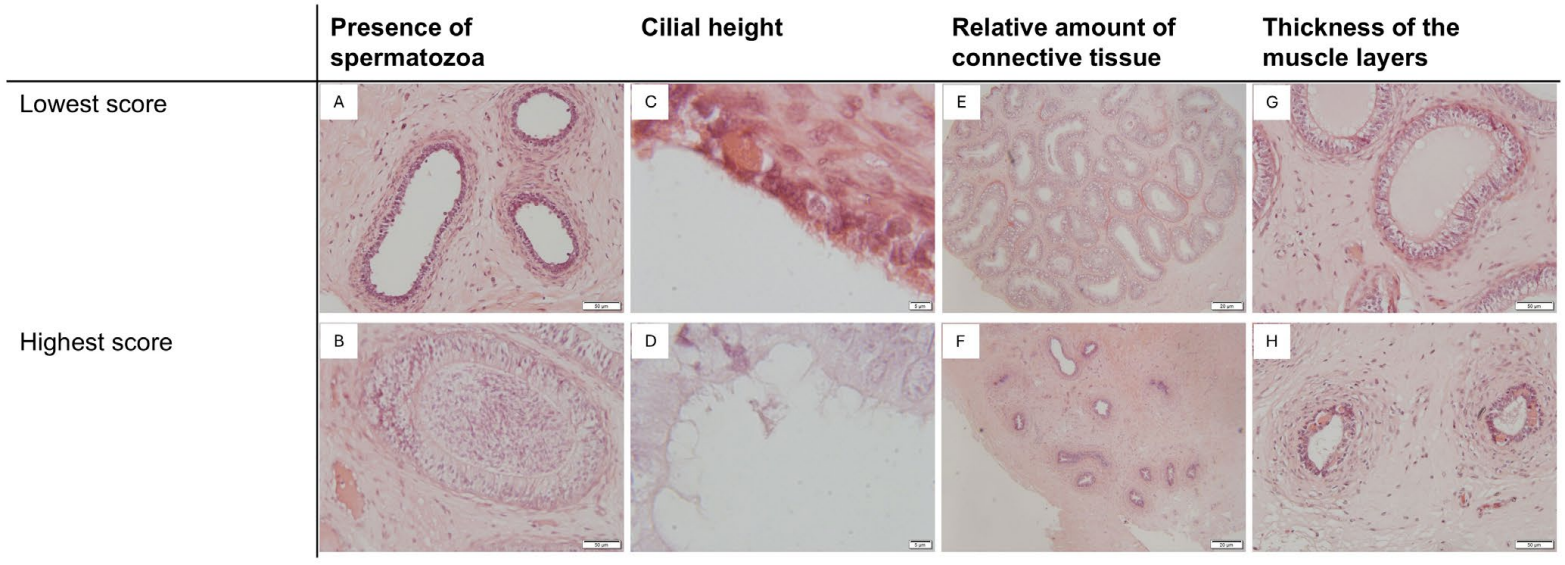
Examples for the lowest (A, C, E, G) and the highest (B, D, F, H) scores of the semiquantitative scoring are given. The presence of spermatozoa (A, B), the cilial height (C, D), the relative amount of connective tissue (E, F) and the thickness of the muscle layers (G, H) are presented.

A scoring system was used for all semiquantitative parameters (Table 1) with examples being presented in Figure 1.

**TABLE 1:** Semiquantitative scoring and applied magnification of the assessed parameters.

Parameters	Magnification	Scoring
Presence of spermatozoa	400×	0 = absence of spermatozoa 1 = few spermatozoa 2 = many spermatozoa
Cilial height	400×	0 = no cilia 1 = low 2 = high
Relative amount of connective tissue	200×	1 = low amount 2 = moderate amount 3 = high amount
Thickness of the muscle layers	200×	1 = low thickness 2 = moderate thickness 3 = high thickness

### 2.4 Statistical Analysis

Microsoft Excel (Version 16.67, 2019, Microsoft Corporation, Washington, DC, USA) was used for data curation, and GraphPad Prism (GraphPad Prism Software 10.0.3, San Diego, CA, USA) for statistical analysis. The Shapiro–Wilk test was applied to test for normal distribution. The body weight was normally distributed; the age was normally distributed following log‐transformation. Consequently, a one‐way ANOVA was applied to test for significant differences regarding body weight and age between groups. After confirming that these parameters did not differ significantly, the target parameters were assessed. The results of these respective parameters were analysed separately for each epididymal segment, namely the epididymal head, body, and tail. The aim was to determine differences between the groups (TG1, TG2, TG3 and CG) and the segments (head, body and tail).

Again, the Shapiro–Wilk test was applied to test for normal distribution and confirmed that all data from the semiquantitative scorings was not normally distributed. Thus, the Kruskal‐Wallis test, followed by the Dunn‐post hoc test, was applied to compare the results between the groups and segments separately. Differently, the results of the epithelial height were normally distributed in all segments. Consequently, an ordinary one‐way ANOVA, followed by Tukey's test was used. The statistical analysis for the total diameter was carried out in the same way (one‐way ANOVA, followed by Tukey's test), except for the head. For the latter, data was not normally distributed resulting in the use of the Kruskal‐Wallis and Dunn‐post hoc test. The correlation between testosterone values and epithelial height and epididymal duct diameter was tested with a Spearman‐correlation, due to not normally distributed data.

Results with *p* < 0.05 were considered statistically significant.

## 3 Results

### 3.1 Animals Included

Mean age, body weight and testosterone concentrations of dogs included in the pre‐defined groups are given in Table 2. Due to the different breeds, a broad variation in body weight and age in CG and TGs was notable, but groups did not differ significantly regarding body weight and age.

**TABLE 2:** Age (in months) and body weight (in kg) of untreated controls (CG) and deslorelin‐treated animals (TG), divided into predefined subgroups (TG1, TG2, TG3)^a^ based on testosterone (*T*) concentrations (ng/mL).

	TG1 (*n* = 10)	TG2 (*n* = 4)	TG3 (*n* = 10)	CG (*n* = 5)
Age (months)	21.4 ± 8.3 (14–36)	25.5 ± 12.4 (12–36)	20.0 ± 5.5 (12–30)	17.6 ± 4.3 (12–24)
Body weight (kg)	22.5 ± 5.6 (15–35)	22.25 ± 3.9 (18–26)	16.6 ± 4.9 (10–24)	20.2 ± 3.1 (16–25)
Testosterone (ng/mL)	0.06 ± 0.02 (0.05–0.1)	0.23 ± 0.05 (0.2–0.3)	2.28 ± 1.16 (0.7–5.2)	3.80 ± 4.77 (0.2–11.9)
Week of castration	0–6	2–10	2–10	n.a.

*Note:* Results are presented as the mean ± standard deviation and the range in parentheses. Additionally, the time (week) of castration is given as the minimum and maximum (range). Groups did not differ in regard to age and body weight.

Abbreviation: n.a., not applicable.

^a^ By definition: TG1: *T* ≤ 0.1 ng/mL; TG2: *T* = 0.2 to 0.5 ng/mL; TG3: *T* ≥ 0.5 ng/mL.

### 3.2 General Histology

The histological assessment of the epididymal segments revealed prominent differences between CG and TGs. In CG, the epididymal duct was characterised by a pseudostratified epithelium with microvilli and a moderate amount of connective tissue. In the lumen, numerous spermatozoa were visible. Alterations in the histology of the TGs included a loss of the epithelial cilia, a reduction of the epithelial height, but an increased relative amount of connective tissue. Moreover, the lumen was devoid of spermatozoa in the vast majority of samples.

### 3.3 Epithelial Height and Epididymal Duct Diameter

**FIGURE 2:**
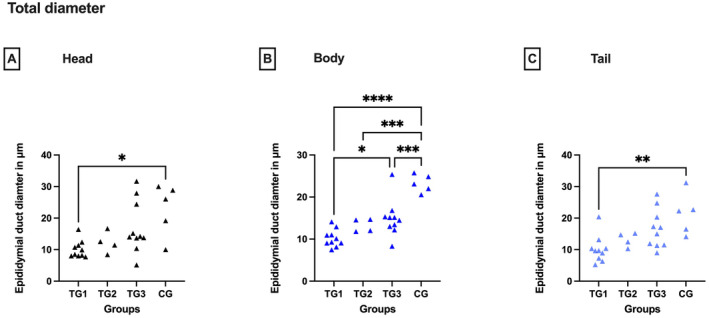
Total diameter of the epididymal duct of the head (A), body (B) and tail (C) of the epididymis. Comparison between the pre‐defined deslorelin‐treated groups (TG1, TG2, TG3)^a^ and the control group (CG). *p < 0.05; **p < 0.01; ***p < 0.001; ****p < 0.0001. ^a^By definition: TG1: *T* ≤ 0.1 ng/mL; TG2: *T* = 0.2 to 0.5 ng/mL; TG3: *T* ≥ 0.5 ng/mL.

**FIGURE 3:**
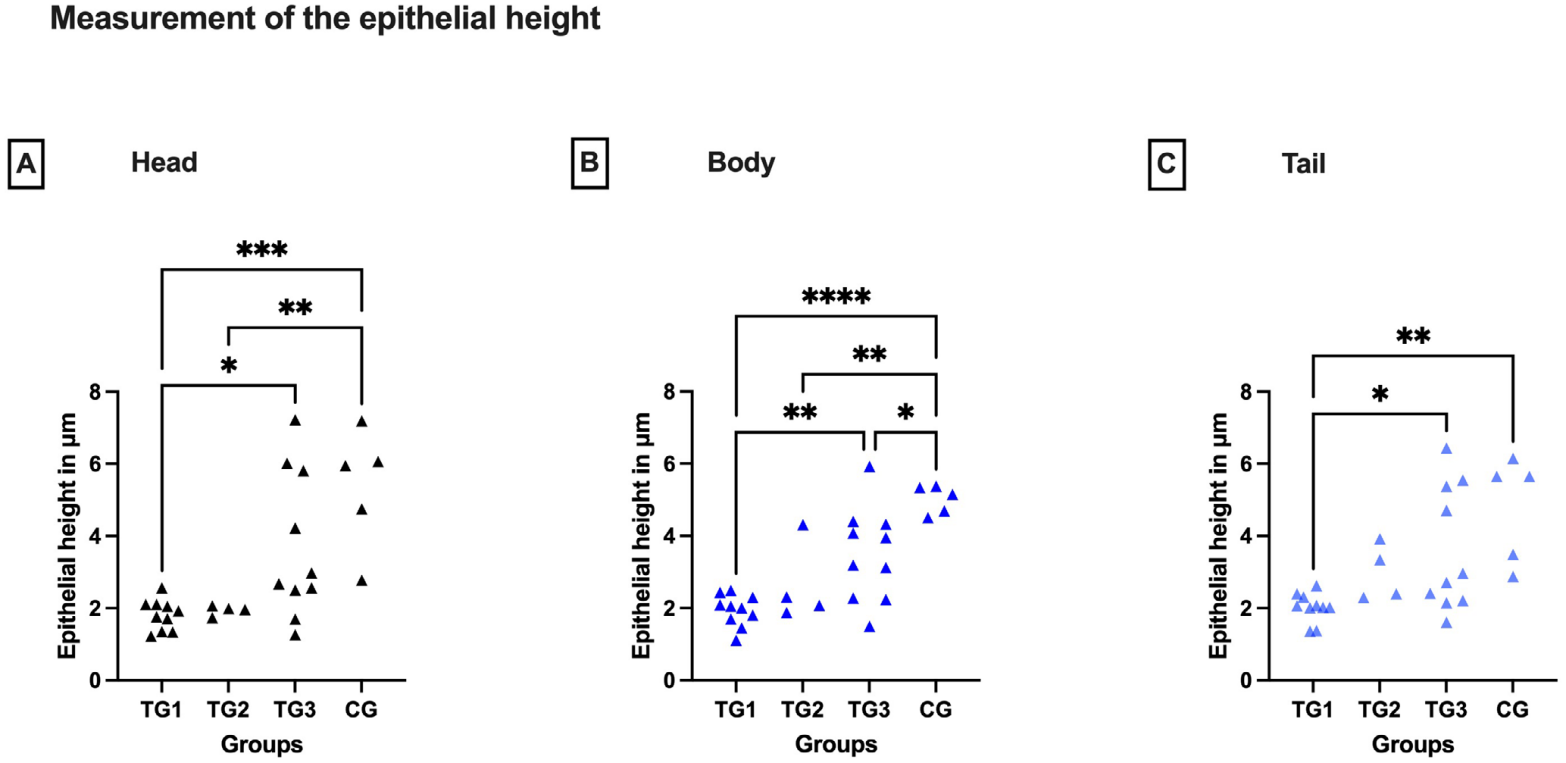
Epithelial height in μm of the head (A), body (B) and tail (C) of the epididymis. Comparison between the pre‐defined deslorelin‐treated groups (TG1, TG2, TG3)^a^ and the control group (CG). *p < 0.05; **p < 0.01; ***p < 0.001; ****p < 0.0001. ^a^By definition: TG1: *T* ≤ 0.1 ng/mL; TG2: *T* = 0.2 to 0.5 ng/mL; TG3: *T* ≥ 0.5 ng/mL.

The total diameter of the epididymal duct was significantly smaller in all segments in TGs than in CG (Kruskal–Wallis: head: *p* < 0.05; ANOVA: body: *p* < 0.0001, tail: *p* < 0.01) (Figure 2). Accordingly, histological assessment of the epithelial height revealed striking differences between the groups independent of the epididymal localisation (ANOVA: head: *p* < 0.001, body: *p* < 0.0001, tail: *p* < 0.005), with the epithelial height being lower in all segments of downregulated animals (Tukey's test: head: *p* < 0.05, body: *p* < 0.0001, tail: *p* < 0.01) (Figure 3). Testosterone values correlated with epithelial height (Spearman correlation coefficient: head: 0.6746; body: 0.8185; tail: 0.8185) and epididymal duct diameter (Spearman correlation coefficient: head: 0.7246; body: 0.7327; tail: 0.5968) with *p* < 0.0001, except the epididymal duct diameter of the tail (*p* = 0.0006).

### 3.4 Presence of Spermatozoa

Spermatozoa were not found in any of the TG1 samples, but exceptionally in TG2 (max. one sample per segment) and TG3 (one to three samples per segment). Different to this, spermatozoa were found in every dog and each segment in CG. Consequently, TG1, 2 and 3 differed significantly from CG (Kruskal–Wallis: *p* < 0.05 to *p* < 0.0001).

### 3.5 Cilial Height

**FIGURE 4:**
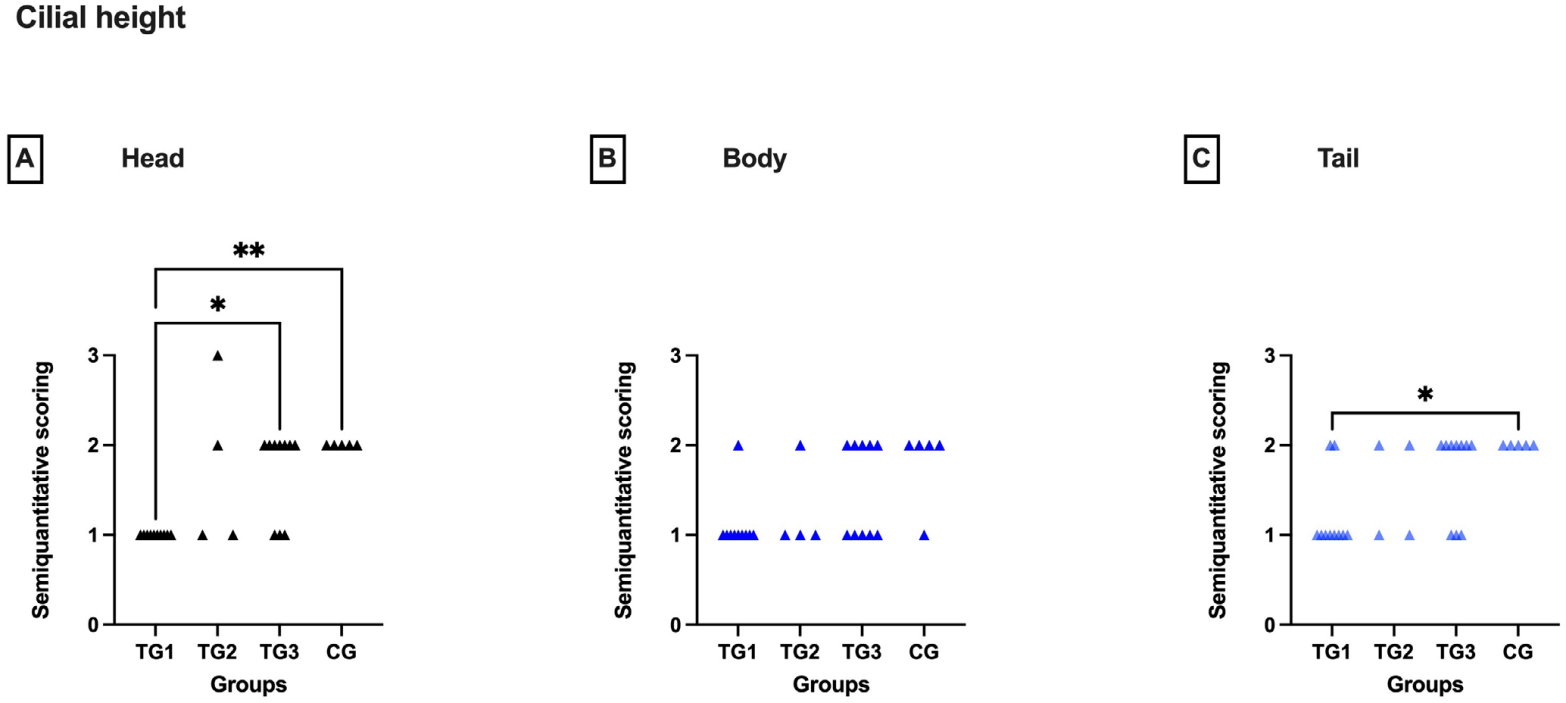
Semiquantitative scoring of the cilial height of the head (A), body (B), and tail (C) of the epididymis. Comparison between the pre‐defined deslorelin‐treated groups (TG1, TG2, TG3)^a^ and the control group (CG). *p < 0.05; **p < 0.01. ^a^By definition: TG1: *T* ≤ 0.1 ng/mL; TG2: *T* = 0.2 to 0.5 ng/mL; TG3: *T* ≥ 0.5 ng/mL.

Cilia were detected in all dogs, independent of segment and group. Nevertheless, different cilial heights were identified between the groups, with higher cilia scores in the CG compared to the TGs. However, differences were only significant in the head and the tail (Kruskal–Wallis: head: *p* < 0.01, tail: *p* < 0.05); in the epididymal body, a trend was recognisable (Kruskal–Wallis: *p* = 0.0526) (Figure 4). In detail, group‐wise comparison revealed lower cilia in TG1 compared to CG (Dunn's test: head and tail: *p* < 0.05, body: *p* = 0.0579) and compared to TG3 in the head only (Dunn's test: *p* < 0.01).

### 3.6 Relative Amount of Connective Tissue

**FIGURE 5:**
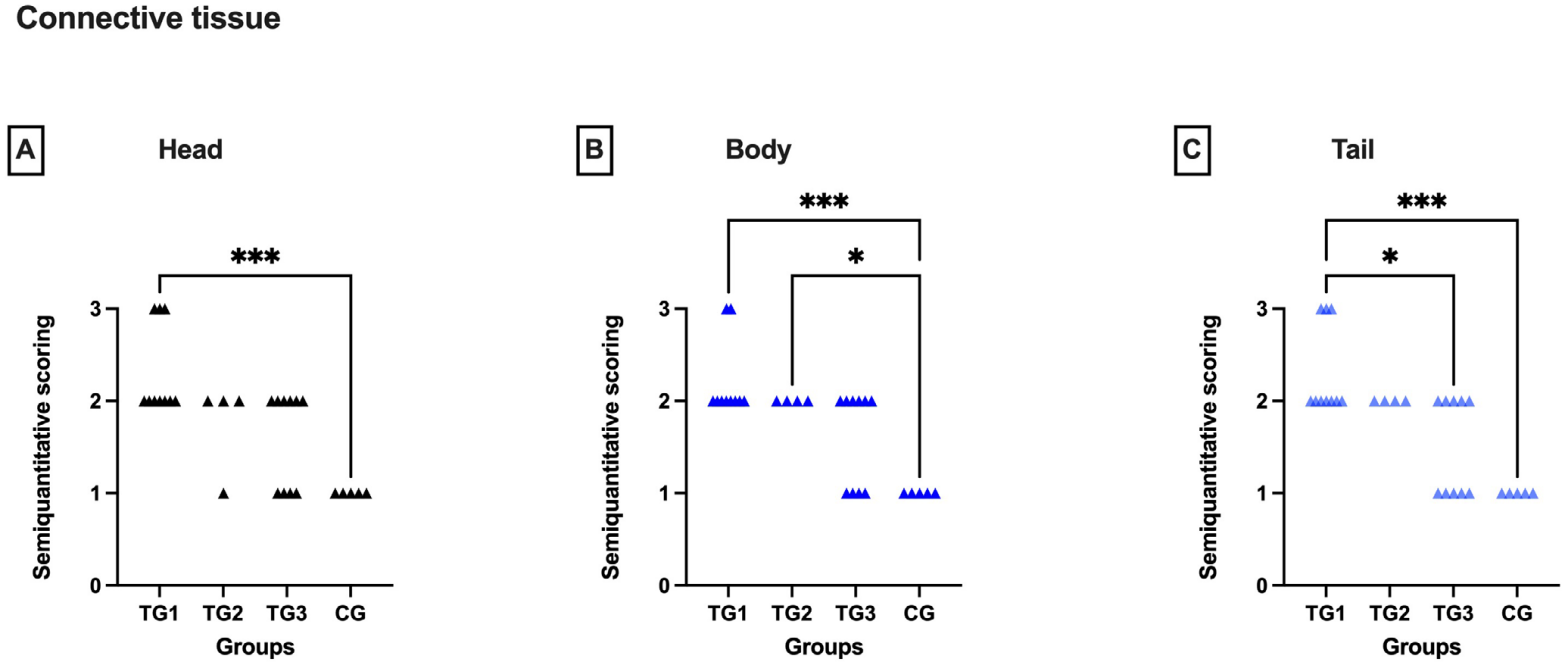
Semiquantitative scoring of the relative amount of connective tissue in the head (A), body (B), and tail (C) of the epididymis. Comparison between the pre‐defined deslorelin‐treated groups (TG1, TG2, TG3)^a^ and control group (CG). *p < 0.05; ***p < 0.001. ^a^By definition: TG1: *T* ≤ 0.1 ng/mL; TG2: *T* = 0.2 to 0.5 ng/mL; TG3: *T* ≥ 0.5 ng/mL.

The relative amount of connective tissue was higher in the TGs when compared to CG (Kruskal Wallis: head: *p* < 0.01, body and tail: *p* < 0.001) (Figure 5) with the relative amount being highest in TG1 compared to CG in all segments (Dunn's test each: *p* < 0.001). For TG2 results differed significantly from CG only in the epididymal body (Dunn's test: *p* < 0.05). Besides, significantly higher scores were obtained in the tail in TG1 compared to TG3 (Dunn's test: *p* < 0.01).

### 3.7 Thickness of the Muscle Layers

**FIGURE 6:**
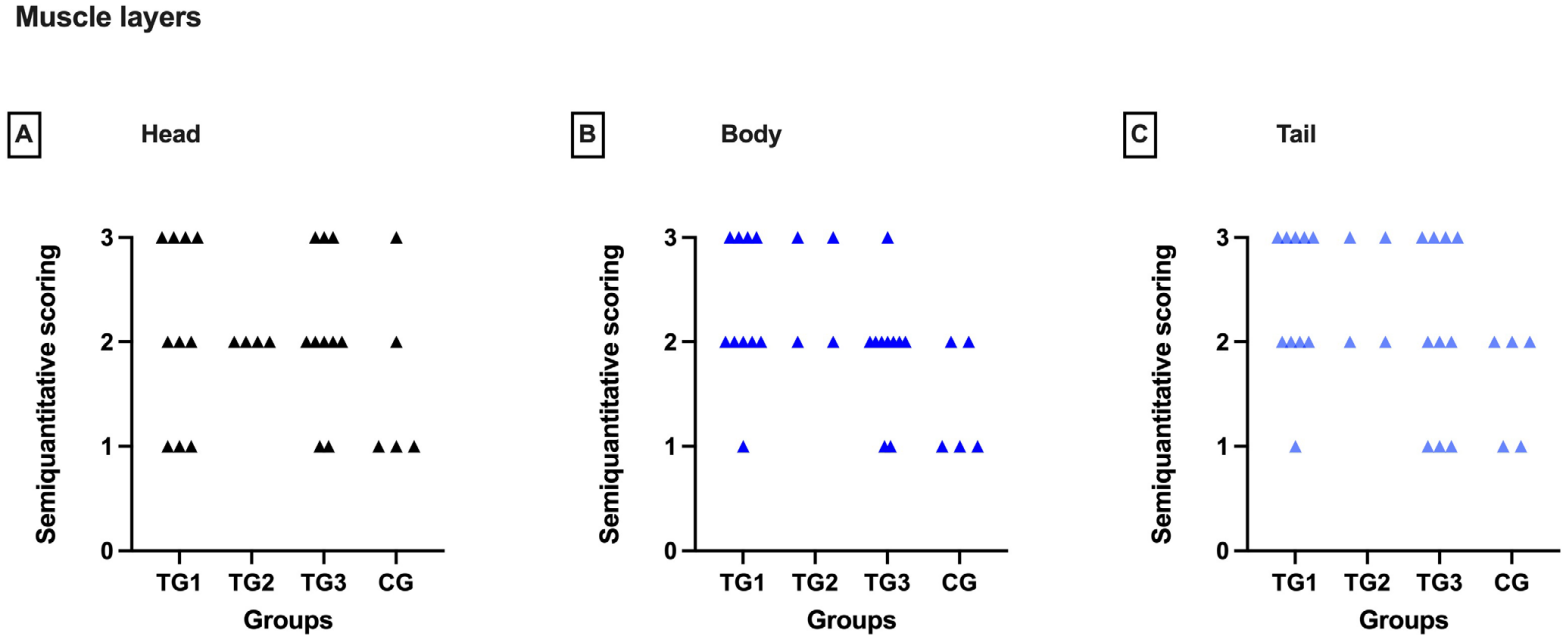
Semiquantitative scoring of the thickness of the muscle layers in the head (A), body (B) and tail (C) of the epididymis. Comparison between the pre‐defined deslorelin‐treated groups (TG1, TG2, TG3)^a^ and control group (CG). Statistical analysis revealed no significant differences. ^a^By definition: TG1: *T* ≤ 0.1 ng/mL; TG2: *T* = 0.2 to 0.5 ng/mL; TG3: *T* ≥ 0.5 ng/mL.

The subjective evaluation of the epididymal muscle layers did not reveal any difference between segments and groups (Figure 6).

## 4 Discussion

Despite the fact that testicular endocrine and germinative function in dogs is well characterised during downregulation and recovery following treatment with GnRH‐agonist SRIs, little is known about the associated changes in the epididymis related to treatment and recovery. Our study, using a selected number of parameters, identified a significant impact of GnRH‐agonist SRI‐associated testosterone withdrawal on epididymal histomorphology. We postulate that the alterations in the investigated parameters significantly impact epididymal function.

The epididymal epithelium has several absorptive and secretory functions (Robaire and Hinton 2015). The epithelial height, but also the tubular diameter were shown to be significantly decreased during effective treatment with the 4.7 mg deslorelin SRI supporting a correlation between presence of testosterone and epididymal function. Similarly, an epididymal epithelial atrophy was described after the use of a 6 mg deslorelin SRI in dogs (Junaidi, Williamson, Trigg, et al. 2009), after orchiectomy in rats (Hamzeh and Robaire 2009), androgen deprivation by orchiectomy or ligation of the extratesticular rete in goats (Goyal et al. 1994), or treatment with GnRH‐antagonists in the chimpanzee (Smithwick and Young 2001). As epithelial height is not only low with basal testosterone (TG1) but increases with increasing testosterone concentrations (TG2/TG3) confirming the identified positive correlation between both parameters, our results clearly support a testosterone dependency of the epididymal epithelial height. It has been well‐described that androgens and androgen withdrawal have significant effects on some epididymal functions, such as contractility (Din‐Udom et al. 1985; Hib and Ponzio 1977), most likely due to changes in cellular signalling or structure. As not only a reduction of epididymal epithelial height but also a reduction of the epididymal duct diameter was observed during basal to low testosterone concentrations, it remains to be clarified whether the reduction in the tubular diameter is related to the absence of spermatozoa, the lower epithelial height or possibly even to duct contraction as testosterone has been shown to cause relaxation (Elfgen et al. 2018). Despite our investigations were restricted to histomorphological characteristics and do not include functional studies, we postulate that androgens are necessary for maintaining the canine epididymal epithelium's function. The observation that the classical androgen receptor, the necessary binding partner for induction of genomic androgen effects, has been identified on epididymal epithelial cells of various species, including the dog (Robaire and Hinton 2015; Younes and Pierrepoint 1981), supports our hypothesis. Nevertheless, further functional studies in the respective model are warranted to confirm an impact on epididymal function.

As mentioned above, increasing testosterone concentrations were associated with increasing epithelial height. Similarly, androgen administration after orchiectomy restored the epididymal epithelium in rats (Robaire and Hamzeh 2011). Nevertheless, and despite only significant in the epididymal body, a larger variation of the epithelial height was observed in all epididymal parts of TG3 compared to CG, indicating that recovery was not completed at the time of castration. This is in good agreement with the confirmed absence of spermatozoa in the lumen in the majority of TG2 and TG3 dogs. Different to this, spermatozoa are found in the lumen of the epididymal duct, especially in the tail, in all animals of CG. As clearly expected from our earlier investigations showing that downregulation is associated with an arrest of spermatogenesis in the testis (Goericke‐Pesch et al. 2009; Stempel et al. 2022a, 2022b) spermatozoa were always absent in the epididymal segment sections of TG1. The fact that spermatozoa were found in one animal in the body and tail in TG2 and three animals (one to all segments, two only in the head) in TG3 clearly support the individual variability of testicular recovery we identified in our earlier studies (Stempel et al. 2022b). The observation that three of the dogs with presence of epididymal spermatozoa were castrated in week 10 after implant removal is in good agreement with the recently reported presence of first spermatozoa in the ejaculate 7 to 10 weeks after the SRI removal (Stempel et al. 2022b). Similar results regarding presence of epididymal spermatozoa have been reported after treatment with a 6 mg deslorelin SRI (Junaidi, Williamson, Martin, et al. 2009). Nevertheless, the last dog with spermatozoa identified in the epididymal duct was castrated only 3 weeks after SRI removal, possibly indicating faster recovery or an alteration of GnRH‐agonist SRI effect before removal.

The cilia play an important role in transporting spermatozoa through the epididymal duct. As such the observed decrease in cilial height in the TGs appears a logical consequence of the lack of sperm related to the sustained deslorelin treatment and was reported earlier (Junaidi, Williamson, Trigg, et al. 2009). Nevertheless, a complete absence of cilia was not detected in any of the dogs or segments in our study, possibly indicating that the cilia fulfil other relevant functions.

The muscle layers around the epididymal duct support the transit of the spermatozoa from the head to the tail of the epididymis. Physiologically, the thickness of the layers in men is known to increase from proximal to more distal regions (Robaire and Hinton 2015). To the best of our knowledge no details have been published for the dog yet. Interestingly, we failed to identify similar trends in dogs, possibly due to small group size and large individual variation.

In conclusion, the GnRH‐agonist SRI treatment and the accompanying testosterone withdrawal caused significant histomorphological changes in all epididymal segments, namely epithelial atrophy, a reduction in cilial height and a relative increase of connective tissue associated with the absence of luminal spermatozoa. In contrast, when testosterone returned to physiological concentrations several weeks after implant removal, histomorphological recovery was observed associated with a predominant lack of significant differences of the investigated morphological parameters compared to healthy untreated controls with normal spermatogenesis. However, the current study did not confirm full reversibility of GnRH‐agonist SRI effects induced on the different histomorphological parameters of the epididymis indicating the need for future studies including later castration time points. Nevertheless, the current results suggest that the epididymis keeps at least partially pace with the development of the testis during the recovery subsequent to GnRH‐agonist SRI treatment and is ready to resume its functions of the male reproductive tract. Further studies at mRNA and protein levels are necessary to gain deeper insights into canine epididymal function during GnRH‐agonist SRI‐associated downregulation and subsequent recovery.

## Author Contributions

Conceptualization, S.G.‐P.; Sample collection, S.As., S.Ay., M.F., G.S. and E.A.; Data curation, H.G. and I.F.; Formal analysis, H.G., H.K., E.‐M.P. and S.G.‐P.; Investigation, H.G., H.K., E.‐M.P. and S.G.‐P.; Methodology, H.K., E.‐M.P. and S.G.‐P.; Resources, S.G.‐P., S.As., S.Ay., M.F. and C.F.; Supervision, S.G.‐P.; Validation, H.G. and S.G.‐P.; Visualization, H.G., E.‐M.P., H.K. and S.G.‐P.; Writing – original draft, H.G., E.‐M.P. and S.G.P.; Writing – review and editing, H.G., E.‐M.P. and S.G.‐P. All authors (H.G., H.K., E.‐M.P., I.F., S.As., S.Ay., M.F., G.S., E.A, C.F. and S.G.‐P.) have read and agreed to the published version of the manuscript.

## Conflicts of Interest

The authors declare the following financial interests/personal relationships which may be considered as potential competing interests: Sandra Goericke‐Pesch reports financial support was provided by Virbac Ltd. including funding grants, lecture fees, and travel reimbursement, besides Sandra Goericke‐Pesch did contract‐based research for Virbac Ltd. Christelle Fontaine is an employee of Virbac, Global Marketing and Market Developement, Carros, France. The funders, however, had no influence on the results. The other authors declare that they have no known competing financial interests or personal relationships that could have appeared to influence the work reported in this paper.

## Data Availability

The data that support the findings of this study are available from the corresponding author upon reasonable request.
